# Screening and diagnostic breast MRI: how do they impact surgical treatment? Insights from the MIPA study

**DOI:** 10.1007/s00330-023-09600-5

**Published:** 2023-05-03

**Authors:** Andrea Cozzi, Giovanni Di Leo, Nehmat Houssami, Fiona J. Gilbert, Thomas H. Helbich, Marina Álvarez Benito, Corinne Balleyguier, Massimo Bazzocchi, Peter Bult, Massimo Calabrese, Julia Camps Herrero, Francesco Cartia, Enrico Cassano, Paola Clauser, Marcos F. de Lima Docema, Catherine Depretto, Valeria Dominelli, Gábor Forrai, Rossano Girometti, Steven E. Harms, Sarah Hilborne, Raffaele Ienzi, Marc B. I. Lobbes, Claudio Losio, Ritse M. Mann, Stefania Montemezzi, Inge-Marie Obdeijn, Umit A. Ozcan, Federica Pediconi, Katja Pinker, Heike Preibsch, José L. Raya Povedano, Carolina Rossi Saccarelli, Daniela Sacchetto, Gianfranco P. Scaperrotta, Margrethe Schlooz, Botond K. Szabó, Donna B. Taylor, Özden S. Ulus, Mireille Van Goethem, Jeroen Veltman, Stefanie Weigel, Evelyn Wenkel, Chiara Zuiani, Francesco Sardanelli

**Affiliations:** 1 Unit of Radiology, IRCCS Policlinico San Donato, Via Rodolfo Morandi 30, 20097 San Donato Milanese, Italy; 2 The Daffodil Centre, Faculty of Medicine and Health, The University of Sydney (Joint Venture with Cancer Council NSW), Sydney, Australia; 3 Department of Radiology, School of Clinical Medicine, Cambridge Biomedical Campus, University of Cambridge, Cambridge, UK; 4 Department of Biomedical Imaging and Image-guided Therapy, Division of Molecular and Structural Preclinical Imaging, Medical University of Vienna, Vienna, Austria; 5 Department of Radiology, Hospital Universitario Reina Sofía, Córdoba, Spain; 6 Department of Radiology, Institut Gustave Roussy, Villejuif, France; 7 BioMaps (UMR1281), INSERM, CEA, CNRS, Université Paris-Saclay, Villejuif, France; 8 Institute of Radiology, Department of Medicine, Ospedale Universitario S. Maria della Misericordia, Università degli Studi di Udine, Udine, Italy; 9 Department of Pathology, Radboud University Medical Center, Nijmegen, The Netherlands; 10 Unit of Oncological and Breast Radiology, IRCCS Ospedale Policlinico San Martino, Genoa, Italy; 11 Department of Radiology, Hospital Universitario de La Ribera, Alzira, Spain; 12 Unit of Breast Imaging, Fondazione IRCCS Istituto Nazionale dei Tumori, Milan, Italy; 13 Breast Imaging Division, IEO, European Institute of Oncology IRCCS, Milan, Italy; 14 Department of Radiology, Hospital Sírio Libanês, São Paulo, Brazil; 15 Department of Radiology, MHEK Teaching Hospital, Semmelweis University, Budapest, Hungary; 16 Breast Center of Northwest Arkansas, Fayetteville, AR USA; 17 Department of Radiology, Di.Bi.MED, Policlinico Universitario Paolo Giaccone, Università degli Studi di Palermo, Palermo, Italy; 18 Department of Radiology and Nuclear Medicine, Maastricht University Medical Center, Maastricht, The Netherlands; 19 Department of Breast Radiology, IRCCS Ospedale San Raffaele, Milan, Italy; 20 Department of Radiology and Nuclear Medicine, Radboud University Medical Center, Nijmegen, The Netherlands; 21 Department of Radiology, The Netherlands Cancer Institute, Amsterdam, The Netherlands; 22 Department of Radiology, Azienda Ospedaliera Universitaria Integrata Verona, Verona, Italy; 23 Department of Radiology and Nuclear Medicine, Erasmus University Medical Center, Rotterdam, The Netherlands; 24 Unit of Radiology, Acıbadem Mehmet Ali Aydınlar University School of Medicine, İstanbul, Turkey; 25 Department of Radiological, Oncological and Pathological Sciences, Università degli Studi di Roma “La Sapienza”, Rome, Italy; 26 Department of Radiology, Breast Imaging Service, Memorial Sloan Kettering Cancer Center, New York, NY USA; 27 Department of Diagnostic and Interventional Radiology, University Hospital of Tübingen, Tübingen, Germany; 28 Kiwifarm S.r.l, La Morra, Italy; 29 Disaster Medicine Service 118, ASL CN1, Saluzzo, Italy; 30 CRIMEDIM, Research Center in Emergency and Disaster Medicine, Università degli Studi del Piemonte Orientale “Amedeo Avogadro”, Novara, Italy; 31 Department of Surgery, Radboud University Medical Center, Nijmegen, The Netherlands; 32 Department of Radiology, Barking Havering and Redbridge University Hospitals NHS Trust, London, UK; 33 Medical School, Faculty of Health and Medical Sciences, The University of Western Australia, Perth, Australia; 34 Department of Radiology, Royal Perth Hospital, Perth, Australia; 35 Gynecological Oncology Unit, Department of Obstetrics and Gynecology, Department of Radiology, Multidisciplinary Breast Clinic, Antwerp University Hospital, University of Antwerp, Antwerpen, Belgium; 36 Maatschap Radiologie Oost-Nederland, Oldenzaal, The Netherlands; 37 Institute of Clinical Radiology and Reference Center for Mammography, University of Münster, Münster, Germany; 38 Department of Radiology, University Hospital of Erlangen, Erlangen, Germany; 39 Department of Biomedical Sciences for Health, Università degli Studi di Milano, Milan, Italy

**Keywords:** Breast-conserving surgery, Breast neoplasms, Magnetic resonance imaging, Mastectomy, Reoperation

## Abstract

**Objectives:**

To report mastectomy and reoperation rates in women who had breast MRI for screening (S-MRI subgroup) or diagnostic (D-MRI subgroup) purposes, using multivariable analysis for investigating the role of MRI referral/nonreferral and other covariates in driving surgical outcomes.

**Methods:**

The MIPA observational study enrolled women aged 18–80 years with newly diagnosed breast cancer destined to have surgery as the primary treatment, in 27 centres worldwide. Mastectomy and reoperation rates were compared using non-parametric tests and multivariable analysis.

**Results:**

A total of 5828 patients entered analysis, 2763 (47.4%) did not undergo MRI (noMRI subgroup) and 3065 underwent MRI (52.6%); of the latter, 2441/3065 (79.7%) underwent MRI with preoperative intent (P-MRI subgroup), 510/3065 (16.6%) D-MRI, and 114/3065 S-MRI (3.7%). The reoperation rate was 10.5% for S-MRI, 8.2% for D-MRI, and 8.5% for P-MRI, while it was 11.7% for noMRI (*p *≤ 0.023 for comparisons with D-MRI and P-MRI). The overall mastectomy rate (first-line mastectomy plus conversions from conserving surgery to mastectomy) was 39.5% for S-MRI, 36.2% for P-MRI, 24.1% for D-MRI, and 18.0% for noMRI. At multivariable analysis, using noMRI as reference, the odds ratios for overall mastectomy were 2.4 (*p *< 0.001) for S-MRI, 1.0 (*p *= 0.957) for D-MRI, and 1.9 (*p *< 0.001) for P-MRI.

**Conclusions:**

Patients from the D-MRI subgroup had the lowest overall mastectomy rate (24.1%) among MRI subgroups and the lowest reoperation rate (8.2%) together with P-MRI (8.5%). This analysis offers an insight into how the initial indication for MRI affects the subsequent surgical treatment of breast cancer.

**Key Points:**

• *Of 3065 breast MRI examinations, 79.7% were performed with preoperative intent (P-MRI), 16.6% were diagnostic (D-MRI), and 3.7% were screening (S-MRI) examinations.*

• *The D-MRI subgroup had the lowest mastectomy rate (24.1%) among MRI subgroups and the lowest reoperation rate (8.2%) together with P-MRI (8.5%).*

• *The S-MRI subgroup had the highest mastectomy rate (39.5%) which aligns with higher-than-average risk in this subgroup, with a reoperation rate (10.5%) not significantly different to that of all other subgroups.*

**Supplementary Information:**

The online version contains supplementary material available at 10.1007/s00330-023-09600-5.

## Introduction

The usefulness of preoperative magnetic resonance imaging (MRI) of the breast, its influence on mastectomy rates, and its potential in reducing reoperation rates are still the focus of heated debates [1–7] and of large studies [8–18]. Meanwhile, in the last 20 years, the routine implementation of breast MRI increased in almost all clinical settings [19–21]. Notably, while MRI screening programs had been established for narrowly defined very high-risk populations [22], the expansion to women with dense breasts [23, 24] has been progressively advocated from 2019 onwards after the results of the DENSE [25, 26] and ECOG-ACRIN EA1141 [27] trials. As a result, an increasing number of breast cancer patients have already undergone breast MRI before diagnosis and before surgical planning: even though these MRI examinations were not performed with preoperative intent, their results ultimately go on to impact treatment.

This was also observed in the Multicenter International Prospective Analysis (MIPA) study, which aimed to compare the mastectomy and reoperation rates between patients who did and did not undergo preoperative breast MRI according to usual practice in 27 centres worldwide [28, 29]. Other MRI indications than preoperative MRI ultimately accounted for more than 20% of patients who underwent MRI, who were excluded from the main analysis which was focused on the preoperative indication [29]. In this 20% cohort, two subgroups could be identified: (i) women with higher-than-average breast cancer risk who had MRI as a screening examination; (ii) women who underwent MRI as a diagnostic examination, mainly for problem-solving purposes.

As the MIPA study enrolled patients from 2013 to 2018, a further increase of the percentage of patients who come to surgical planning with screening or diagnostic MRI beyond the aforementioned 20% is easily foreseeable or—most likely—already occurring, but the effects of MRI in these subgroups on the choice between breast-conserving surgery (BCS) and mastectomy remain to be ascertained.

Therefore, this report from the MIPA study will address mastectomy rates and reoperation rates in women who underwent breast MRI for screening and diagnostic purposes, using multivariable analysis for investigating the role of MRI referral/nonreferral and other covariates in driving surgical outcomes.

## Materials and methods

Details on the design and methods of the MIPA study have been previously reported [28, 29] and are summarised here. The MIPA study observationally enrolled women aged 18–80 years with newly diagnosed breast cancer at core-needle or vacuum-assisted biopsy (CNB/VAB), without indications for neoadjuvant therapy and amenable to upfront surgery. Following routine practice of each centre, the diagnostic pathway included a variable combination of the following: conventional imaging, i.e. mammography and/or breast ultrasonography; stereotactic, ultrasound- or MRI-guided CNB/VAB; bilateral contrast-enhanced MRI; and eventual further CNB/VAB sampling of additional lesions detected by preoperative MRI. Data from all these steps were recorded alongside data from surgical planning stages (multidisciplinary team meetings or direct interview with clinicians) and from surgical pathology.

### Patient subgroups

Purpose and timing of MRI referral were recorded and classified as “screening”, “diagnostic”, or “preoperative”, specific purposes behind MRI referral in the first two subgroups being detailed in Table 1. Notably, screening and diagnostic MRI had to be performed before CNB/VAB. This work extends the per-protocol and patient-based analysis of surgical endpoints (detailed below), already performed for patients who did not undergo MRI (noMRI subgroup) and patients who underwent MRI with a preoperative purpose (P-MRI subgroup) [29], to patients from the screening (S-MRI) and diagnostic (D-MRI) subgroups.

**Table 1:** Referral purposes among the S-MRI and D-MRI subgroups

MRI subgroup	Reason behind MRI referral	*N*	%
Diagnostic (D-MRI)	Equivocal finding at mammography and/or ultrasonography	425	83.3%
	Contralateral breast screening	17	3.3%
	Nipple discharge	14	2.8%
	Pre-biopsy staging and lesion size evaluation	12	2.4%
	Request by surgeon/oncologist	8	1.6%
	Carcinoma of unknown primary origin	6	1.2%
	Palpable lesion	6	1.2%
	Breast implant evaluation	1	0.1%
	*Unspecified*	*21*	*4.1%*
	**Subgroup total**	**510**	**100%**
Screening (S-MRI)	High risk	72	63.2%
	Intermediate risk	24	21.0%
	Average risk	18	15.8%
	**Subgroup total**	**114**	**100%**

*MRI*, magnetic resonance imaging

Evaluation of surgical planning and outcomes was recorded at four different timepoints: (1) surgical planning according to findings from conventional imaging only; (2) surgical planning according to findings from conventional imaging and MRI; (3) actual surgery performed; (4) surgical outcome and immediate/short-term reoperation for close or positive margins. While data from all four timepoints were available for the P-MRI subgroup, timepoint 1 was not available for the S-MRI and D-MRI, as MRI was embedded in the diagnostic process and no surgical treatment was planned before the patient underwent MRI. Therefore, while analyses on timepoints 3 and 4 were conducted as planned, timepoints 1 and 2 were collapsed into a single timepoint, i.e. “surgical planning after imaging”, considering surgical planning after MRI for the S-MRI, D-MRI, and P-MRI subgroups and surgical planning after conventional imaging for the noMRI subgroup.

### Endpoints

The two primary surgical endpoints are first-line mastectomy and immediate/short-term reoperation for close or positive margins. The two secondary surgical endpoints are first-line bilateral mastectomy and the overall mastectomy rate, the latter obtained adding first-line mastectomies to conversions from BCS to mastectomy after reoperation.

### Data analysis

**Fig. 1:**
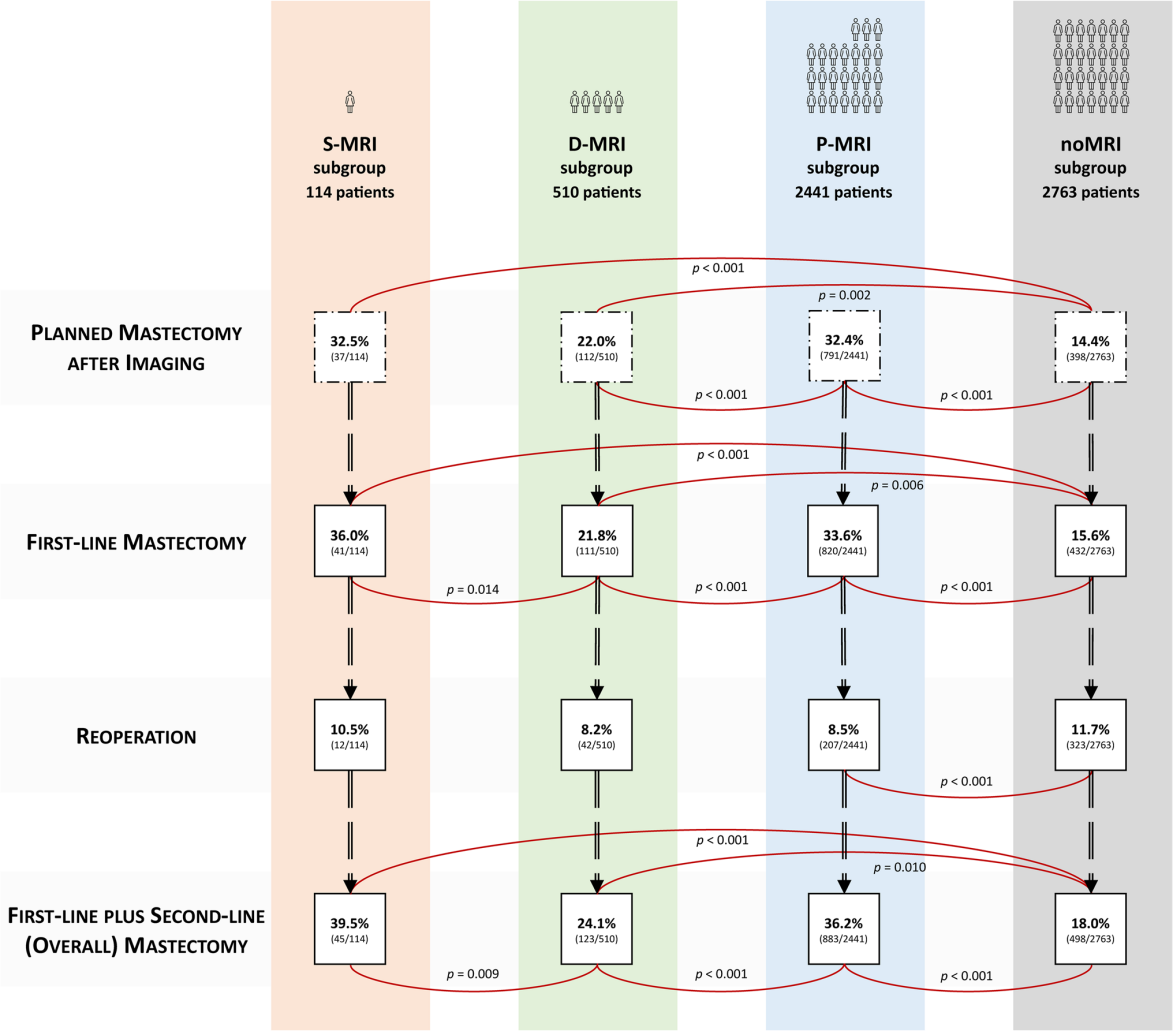
Stage-by-stage analysis of surgical endpoints in the four subgroups. Red lines indicate comparisons of rates between subgroups that were statistically significant at post hoc testing after adjustment with the Bonferroni-Holm correction (adjusted *p* values are shown). S-MRI, screening magnetic resonance imaging; D-MRI, diagnostic magnetic resonance imaging; P-MRI, preoperative magnetic resonance imaging

The Supplementary Material details univariate comparisons of demographic, imaging, CNB/VAB, and surgical pathology characteristics among the noMRI, S-MRI, D-MRI, and P-MRI subgroups. Figure 1 presents univariate comparisons of surgical planning and outcomes. Considering the observational and non-randomised design of the MIPA study and size imbalances between the four subgroups, non-parametric statistics were used, namely the *χ*^2^ and Fisher’s tests for categorical variables and the Kruskal-Wallis test for continuous variables. To account for multiple testing, the Bonferroni correction was applied for the 24 overall comparisons—resulting in a *p* < 0.002 threshold for statistical significance—whereas *p* values presented in pairwise post hoc testing were automatically adjusted with the Bonferroni-Holm correction (adjusted *p* < 0.05 significance threshold).

Multivariable binary logistic regression analysis was then performed to estimate the relative effect of covariates on the four outcomes of interest by calculating adjusted odds ratios (ORs) for such predictors with their 95% confidence intervals (CIs). Covariate selection from the clinically reasoned pool of demographic, imaging, and pathology variables was performed for each of the four binary logistic regressions using stepwise multivariable linear regression (forward selection with *p* < 0.1 as the threshold for variable inclusion) and is detailed in the Supplementary Material.

All analyses were performed with SPSS v.26.0 (IBM SPSS Inc.).

## Results

### Study population

Of the 7245 patients enrolled between June 2013 and November 2018, 1417 were excluded due to unretrievable or missing data. Thus, 5828 patients entered this analysis with 2763 (47.4%) in the noMRI subgroup. Among the 3065 (52.6%) patients who underwent MRI, 2441 (79.7%) were in the P-MRI subgroup, 510 (16.6%) in the D-MRI subgroup, and 114 (3.7%) in the S-MRI subgroup.

As detailed in Tables E1, E2, E3, and E4 (Supplementary Material), differences between MRI subgroups in demographic, imaging, and pathology characteristics became evident when examining the S-MRI and D-MRI subgroups. Patients of the S-MRI subgroup had different characteristics in almost all analysed indicators, being younger than those of the P-MRI subgroup, having a far higher proportion of familial or personal genetically proven increased risk of breast cancer and the highest rates of small and single-focus cancers. The D-MRI subgroup exhibited an intermediate profile either between the noMRI and the P-MRI subgroups (e.g. in terms of lesion size and focality at conventional imaging and final pathology) or between the S-MRI and the P-MRI subgroups (e.g. in terms of patients’ age, hormonal status, lesion size and focality at MRI, rate of invasive lobular component at CNB/VAB and surgical pathology).

**Table 2:** Multivariable binary logistic regression model of variables associated with first-line mastectomy

		*p* value	Odds ratio	95% CI
MRI referral	noMRI	Reference	–	–
	Screening MRI	< 0.001	2.5	1.5–4.0
	Diagnostic MRI	0.300	1.2	0.9–1.5
	Preoperative MRI	< 0.001	2.2	1.9–2.5
Breast density	ACR BI-RADS class *a*	Reference	–	–
	ACR BI-RADS class *b*	0.498	1.1	0.9–1.4
	ACR BI-RADS class *c*	0.168	1.2	0.9–1.5
	ACR BI-RADS class *d*	0.001	1.7	1.2–2.3
Breast cancer risk	Familial breast cancer risk	0.012	2.0	1.2–3.3
Hormonal status	Postmenopausal	Reference	–	–
	Premenopausal	< 0.001	1.6	1.4–1.9
	Perimenopausal	0.247	1.2	0.9–1.5
Lesion focus at DM	No observable lesion	Reference	–	–
	Single focus	< 0.001	0.7	0.5–0.8
	Multifocal	0.211	1.2	0.9–1.7
	Multicentric	0.001	2.4	1.4–4.2
Largest lesion diameter at DM	≥ 20 mm	< 0.001	2.0	1.7–2.4
Lesion focus at US	No observable lesion	Reference	–	–
	Single focus	0.336	0.9	0.7–1.1
	Multifocal	< 0.001	2.4	1.8–3.3
	Multicentric	< 0.001	5.8	3.2–10.8
Largest lesion diameter at US	≥ 20 mm	< 0.001	2.1	1.7–2.5
Core-needle or vacuum-assisted biopsy	Pure DCIS	0.196	1.1	0.9–1.4
	Lobular component	< 0.001	1.7	1.4–2.1

*CI*, confidence interval; *ACR BI-RADS*, American College of Radiology Breast Imaging Data and Reporting System; *DM*, digital mammography; *US*, ultrasonography; *MRI*, magnetic resonance imaging; *DCIS*, ductal carcinoma in situ

**Table 3:** Multivariable binary logistic regression model of variables associated with bilateral first-line mastectomy

		*p* value	Odds ratio	95% CI
MRI referral	noMRI	Reference	–	–
	Screening MRI	< 0.001	29.2	12.6–67.6
	Diagnostic MRI	0.004	3.6	1.5–8.7
	Preoperative MRI	< 0.001	6.8	3.7–12.5
Breast cancer risk	Familial breast cancer risk	0.001	3.9	1.8–8.5
Hormonal status	Postmenopausal	Reference	–	–
	Premenopausal	0.079	1.4	1.0–2.1
	Perimenopausal	0.376	1.3	0.7–2.4
Lesion features at US	No observable lesion	Reference	–	–
	Single focus	0.140	1.5	0.9–2.7
	Multifocal	0.010	2.5	1.2–5.0
	Multicentric	< 0.001	7.5	3.5–16.4
Core-needle or vacuum-assisted biopsy	Lobular component	0.001	2.1	1.4–3.3

*CI*, confidence interval; *US*, ultrasonography; *MRI*, magnetic resonance imaging

**Table 4:** Multivariable binary logistic regression model of variables associated with reoperation for close or positive margins

		*p* value	Odds ratio	95% CI
MRI referral	noMRI	Reference	–	–
	Screening MRI	0.452	0.8	0.4–1.5
	Diagnostic MRI	0.029	0.7	0.5–1.0
	Preoperative MRI	< 0.001	0.7	0.6–0.8
Hormonal status	Postmenopausal	Reference	–	–
	Premenopausal	0.021	0.8	0.6–1.0
	Perimenopausal	0.246	0.8	0.6–1.1
Lesion focus at US	No observable lesion	Reference	–	–
	Single focus	0.016	0.8	0.6–1.0
	Multifocal	0.069	0.7	0.5–1.0
	Multicentric	0.029	0.4	0.2–0.9
Largest lesion diameter at US	≥ 20 mm	0.029	0.8	0.6–1.0
Surgical pathology	Pure DCIS	< 0.001	2.5	1.9–3.3
	DCIS associated to invasive cancer	< 0.001	1.8	1.4–2.2
	Largest lesion diameter ≥ 20 mm	< 0.001	1.5	1.2–1.8
	Multifocal or multicentric cancer	0.001	1.5	1.2–1.9
	Lobular component	0.017	1.4	1.1–1.8

*CI*, confidence interval; *US*, ultrasonography; *MRI*, magnetic resonance imaging; *DCIS*, ductal carcinoma in situ

### Surgical endpoints

After imaging, the planned mastectomy rate was lowest in the noMRI subgroup (14.4%, 398/2763 patients, *p *< 0.001) and highest in the S-MRI (32.5%, 37/114 patients) and P-MRI subgroups (32.4%, 791/2441 patients). The D-MRI subgroup had an intermediate rate of 22.0% (112/510 patients, adjusted *p* values < 0.001 for pairwise comparisons).

#### First-line mastectomy

These trends were confirmed analysing the rates of actually performed first-line mastectomy. Except for the S-MRI subgroup, actually performed mastectomy rates only slightly differed from initial planning, due to patient preference-based conversions of planned BCS to mastectomy and viceversa. The S-MRI subgroup had the highest mastectomy rate (36.0%, 41/114 patients), followed by the P-MRI subgroup (33.6%, 820/2441 patients), the D-MRI subgroup (21.8%, 111/510 patients), and the noMRI subgroup (15.6%, 432/2763).

The findings from univariate analysis were confirmed by multivariable linear (Table E5) and binary logistic (Table 2) regressions, where S-MRI had the highest significant OR for first-line mastectomy (2.5, *p *< 0.001), D-MRI having the lowest and non-significant OR (1.2, *p *= 0.300). Multicentric cancer diagnosis at ultrasonography (OR 5.8, *p *< 0.001) or mammography (OR 2.4, *p *= 0.001) was the imaging feature more strongly associated with mastectomy, while among CNB/VAB features the presence of invasive lobular carcinoma carried a 1.7 OR for mastectomy (*p *< 0.001).

**Table 5:** Multivariable binary logistic regression model of variables associated with overall mastectomy

		*p* value	Odds ratio	95% CI
MRI referral	noMRI	Reference	–	–
	Screening MRI	< 0.001	2.4	1.5–3.8
	Diagnostic MRI	0.957	1.0	0.8–1.3
	Preoperative MRI	< 0.001	1.9	1.6–2.2
Breast density	ACR BI-RADS class *a*	Reference	–	–
	ACR BI-RADS class *b*	0.081	1.2	1.0–1.5
	ACR BI-RADS class *c*	0.017	1.3	1.1–1.7
	ACR BI-RADS class *d*	< 0.001	1.8	1.3–2.5
Breast cancer risk	Familial breast cancer risk	0.003	2.2	1.3–3.7
Hormonal status	Postmenopausal	Reference	–	–
	Premenopausal	< 0.001	1.6	1.4–1.9
	Perimenopausal	0.434	1.1	0.9–1.4
Lesion focus at DM	No observable lesion	Reference	–	–
	Single focus	0.005	0.7	0.6–0.9
	Multifocal	0.578	1.1	0.8–1.5
	Multicentric	0.002	2.5	1.4–4.5
Largest lesion diameter at DM	≥ 20 mm	< 0.001	1.8	1.5–2.1
Lesion focus at US	No observable lesion	Reference	–	–
	Single focus	0.194	0.9	0.7–1.1
	Multifocal	< 0.001	1.8	1.3–2.4
	Multicentric	< 0.001	4.2	2.1–8.2
Largest lesion diameter at US	≥ 20 mm	< 0.001	1.8	1.5–2.2
Core**-**needle or vacuum**-**assisted biopsy	Pure DCIS	< 0.001	1.7	1.3–2.2
	Lobular component	< 0.001	1.5	1.2–1.9
Surgical pathology	Pure DCIS	< 0.001	0.6	0.4–0.8
	DCIS associated to invasive cancer	0.005	0.8	0.7–0.9
	Largest lesion size ≥ 20 mm	< 0.001	1.8	1.5–2.1
	Multifocal or multicentric cancer	< 0.001	3.9	3.2–4.7

*CI*, confidence interval; *ACR BI-RADS*, American College of Radiology Breast Imaging Data and Reporting System; *DM*, digital mammography; *US*, ultrasonography; *MRI*, magnetic resonance imaging; *DCIS*, ductal carcinoma in situ

The S-MRI subgroup had the highest rate of bilateral mastectomy among patients undergoing first-line mastectomy (34.1%, 14/41 patients, adjusted *p* values < 0.001 for all comparisons), followed by P-MRI (10.6%, 87/820 patients) and D-MRI (8.1%, 9/111 patients), whereas only 12/432 first-line mastectomies (2.8%) in the noMRI subgroup were bilateral. Multivariable regression (Table E6 and Table 3) mirrored the clinical bias towards bilateral mastectomy in patients undergoing screening MRI (OR 29.2, *p *< 0.001) or patients with multicentric cancer diagnosis at ultrasonography (OR 7.5, *p *< 0.001).

#### Reoperation

The noMRI subgroup had the highest reoperation rate for close or positive margins (11.7%, 323/2763 patients), followed by the S-MRI subgroup (10.5%, 12/114 patients), the P-MRI subgroup (8.5%, 207/2441 patients), and the D-MRI subgroup with the lowest reoperation rate (8.2%, 42/510 patients). While a significant difference (adjusted *p* value < 0.001) was found between the reoperation rates of the noMRI and P-MRI subgroups, no significant difference was observed between different MRI subgroups (adjusted *p* values = 1.000).

The S-MRI subgroup had the highest rate of BCS that underwent reoperation with conversion to mastectomy instead of wider local excision (4/11, 36.4%), followed by the P-MRI subgroup (63/190, 33.2%), the D-MRI subgroup (12/40, 30.0%), and the noMRI subgroup (66/316, 20.9%). A significant overall difference (*p *= 0.016) was observed, the pairwise comparison between the P-MRI and the noMRI subgroup showing the only significant difference (adjusted *p* value = 0.013, all other comparisons *p *= 1.000).

Multivariable modelling (Table E7 and Table 4) highlighted how all indications for MRI had a protective effect against reoperation (OR 0.7 with *p* values ≤ 0.029 for D-MRI and P-MRI, OR 0.8 with *p *= 0.452 for S-MRI), a role shared with many drivers of first-line mastectomy such as multicentric cancer presentation (OR 0.4, *p *= 0.029) and lesion size ≥ 20 mm (OR 0.8, *p *= 0.029) at ultrasonography. All factors associated with re-operations came from pathology, with the presence of pure DCIS carrying the highest OR (2.5, *p *< 0.001) for reoperation, followed by the presence of DCIS associated with invasive cancer (OR 1.8, *p *< 0.001).

#### Overall mastectomy rate

Differences outlined in previous comparisons were more evident in overall mastectomy rates: this rate rose to 39.5% (45/114 patients) in the S-MRI subgroup, closely followed by the P-MRI subgroup (36.2%, 883/2441 patients, pairwise comparison adjusted *p *= 1.000). Compared to these two subgroups, as was for first-line surgery, the noMRI subgroup had half (or less) of these rates, with an 18.0% overall mastectomy rate (498/2763 patients, adjusted *p* values < 0.001 for the comparisons with S-MRI and P-MRI). The 24.1% overall mastectomy rate in the D-MRI subgroup (123/510 patients) significantly differed from all subgroups while being marginally closer to the noMRI subgroup (adjusted *p* values of 0.009, 0.001, and 0.010 for the comparisons with the S-MRI, P-MRI, and noMRI subgroups, respectively). The multivariable model (Table E8 and Table 5) showed how variables strongly associated with reoperation protected against overall mastectomy, such as pure DCIS (OR 0.6, *p *< 0.001) or DCIS associated with invasive cancer (OR 0.8, *p *= 0.005) at surgical pathology and single-focus cancer presentation at mammography (OR 0.7, *p *= 0.005). Conversely, strong association with overall mastectomy was observed for surgical pathology findings that implied a reoperation strategy based on mastectomy, such as multifocal or multicentric cancer (OR 3.9, *p *< 0.001) and lesion size ≥ 20 mm (OR 1.8, *p *< 0.001). Clinical variables driving upfront mastectomy were also associated with overall mastectomy, such as multicentric cancer at conventional imaging (OR 2.5 with *p *= 0.002 for mammography, OR 4.2 with *p *< 0.001 for ultrasonography), screening MRI referral (OR 2.4, *p *< 0.001), high familial risk (OR 2.2, *p *= 0.003), and pure DCIS or invasive lobular carcinoma diagnosis at CNB/VAB (OR 1.7 with *p *< 0.001 and OR 1.5 with *p *< 0.001, respectively).

## Discussion

The rationale of this subgroup analysis of the MIPA study was based on the observation that 21% of women in the MRI subgroup had already undergone MRI for screening or diagnostic purposes when they reached treatment planning. In these patients, the MRI result is used in surgical decision-making, bypassing the debate about the appropriateness of its preoperative use [5, 7]. Besides concerns about overdiagnosis and overtreatment, this represents a relevant issue caused by the ever-widening application of MRI from problem-solving [30] to screening [23].

As expected, the characteristics of the S-MRI subgroup translated in three times higher bilateral mastectomy rates compared to the D-MRI and P-MRI subgroups, both at surgical planning after imaging and at the evaluation of actually performed first-line surgery, also acknowledging trends towards contralateral prophylactic mastectomy [31, 32] and the influence of patient preferences on 9.8% of all mastectomies (compared to “only” 3.7% in the P-MRI subgroup). Of note, while the reoperation rate of 10.5% in the S-MRI subgroup was higher than that in the D-MRI and P-MRI subgroups (8.2% and 8.5%, respectively) and was close to that in the noMRI subgroup (11.7%), re-operations were performed in 92% of cases on previously attempted BCS.

In the D-MRI subgroup, MRI referral was due to equivocal findings at conventional imaging in 83% of cases (MRI as problem solving), with a patient profile in-between the noMRI and the P-MRI subgroups. In the latter, the composite selection bias towards MRI [6, 18] defined a subgroup of younger patients with complex cases, larger lesions, and higher rates of multifocal or even multicentric disease. Conversely, this phenomenon was less conspicuous in the D-MRI subgroup, likely because cancers exhibiting equivocal findings at conventional imaging come from the whole spectrum of breast cancer stages, as recently shown by a population-based study on problem-solving MRI [33]. Univariate analysis highlighted how the intermediate profile of the D-MRI subgroup extended to surgical endpoints, with the lowest rates of first-line and overall mastectomy among MRI subgroups, and the lowest reoperation rate in the whole study (8.2%). These findings were confirmed by multivariable analysis, where patients’ association to the D-MRI subgroup had the lowest OR for first-line mastectomy, first-line bilateral mastectomy, and overall mastectomy. Notably, D-MRI carried a 1.0 OR of overall mastectomy (95% CI 0.8–1.3) compared to the noMRI subgroup.

The comparison of the D-MRI and P-MRI subgroups provided other insights: P-MRI examinations were driven by the sole purposes of ipsilateral staging and contralateral screening in CNB/VAB-proven cancer, whereas the spectrum of indications in the D-MRI subgroup included the heterogeneous “equivocal findings” at conventional imaging and a substantial diversity in the remaining 17% of cases. Notably, the MIPA study did not include patients without breast cancer. Thus, the D-MRI subgroup was relatively small in size due to the fact that only 10–13% of patients undergoing problem-solving MRI are reported to be ultimately diagnosed with breast cancer [33, 34]. Still, the imaging and pathology profiles of cancers in the D-MRI subgroup in our study seem to be less polarised towards large and multifocal or multicentric tumours compared to cancers from the P-MRI subgroup, as already observed in a population-based study [33].

Moreover, multivariable regression analysis indicated that first-line mastectomy, reoperation, and overall mastectomy were driven by patient-specific imaging and pathology features rather than by the indication for the MRI examination. These findings hint that overtreatment concerns might be less pronounced than what could be surmised by the experience of MRI screening in high-risk populations—that naturally carry a strong multi-layered propensity towards surgery [35]—or by the experience with preoperative MRI, solely performed on CNB/VAB-proven cancers. Indeed, the D-MRI subgroup has a far more “neutral” purpose behind MRI referral, while the S-MRI and P-MRI subgroups are affected by referral biases that—albeit different—ultimately drive surgical planning towards mastectomy. These biases are supported by patient-based, imaging-based, and biopsy-based characteristics that, respectively, characterise the S-MRI and P-MRI subgroups in comparison to the noMRI and D-MRI subgroups. For example, while the use of MRI in the S-MRI subgroup could be still considered a “diagnostic” one, as in the D-MRI subgroup, data from Tables E1, E2, and E3 highlight how factors such as age and familial or personal genetically proven increased risk of breast cancer characterise this subgroup and constitute an a priori referral bias towards mastectomy. Evidence from follow-up and secondary analyses of breast MRI screening trials outside the high-risk setting will therefore be crucial to better define these issues, also acknowledging that the panorama of contrast-enhanced breast imaging saw an extension towards contrast-enhanced mammography [36], which has already received a conditional recommendation from the European Commission Initiative on Breast Cancer to substitute MRI in the preoperative setting [37], even before the completion of specific randomised trials [38]. Conversely, the P-MRI subgroups carry an a posteriori referral bias, supported by several characteristics that only emerge when the diagnostic pathway has already begun, i.e. imaging-based and biopsy-based features such as larger maximal lesion diameters and higher rates of multifocal or multicentric presentation at conventional imaging, and the presence of lobular component at CNB/VAB.

Limitations of this work and of the MIPA study itself chiefly reside in its non-randomised and observational design and in the impossibility of conducting a thorough evaluation of factors that affect surgical decision-making, such as individual surgeon experience and choice, access to advanced reconstruction techniques, or patient-specific and institutional factors. Furthermore, considering the 2013–2018 enrolment timeframe, the potential intervening effect of three factors that emerged in the last decade must be acknowledged: first, the technical and clinical improvements of breast MRI; second, the expanded role of MRI compared to the guidelines issued at the beginning of the 2010s [19]; third, the widespread adoption of digital breast tomosynthesis. These factors could have mitigated the imbalance between subgroups.

In conclusion, this subgroup analysis of the MIPA study confirmed that in all patients, rather than the different reasons for an MRI referral, many other factors drove surgical planning, including demographic, conventional imaging, and pathologic features. Patients with MRI performed before CNB/VAB for screening or diagnostic purposes had different characteristics and surgical outcomes compared to both the noMRI subgroup and the P-MRI subgroup. Patients from the D-MRI subgroup had the lowest overall mastectomy rate (24.1%) among MRI subgroups and the lowest absolute reoperation rate (8.2%) together with the P-MRI subgroup (8.5%). This analysis offers an insight into how the initial indication for the MRI affects the subsequent influence on surgical treatment of breast cancer.

## Supplementary Information

Below is the link to the electronic supplementary material.

Supplementary file1 (PDF 285 KB)

## Abbreviations

BCS: Breast-conserving surgery
CI: Confidence interval
DCIS: Ductal carcinoma in situ
MIPA: Multicenter International Prospective Analysis
MRI: Magnetic resonance imaging
OR: Odds ratio

## Declarations

### Guarantor

The scientific guarantor of this publication is Prof. Francesco Sardanelli, MD.

### Statistics and biometry

The first three authors (A.C., G.D.L, N.H.) have significant statistical expertise.

### Study subjects or cohorts overlap

Two study subgroups (patients who underwent MRI with preoperative intent and patients who did not undergo MRI, for a total of 5204 patients) were already included in the main paper of the MIPA study results (Sardanelli et al, European Radiology 2022; 10.1007/s00330-021-08240-x) and are used in this paper in a comparative analysis with patients from two other subgroups that are first reported here (patients who underwent screening MRI and patients who underwent diagnostic/problem-solving MRI).

### Methodology

• Prospective

• Observational

• Multicentre study

## References

[1] Morrow M (2009). Should routine breast cancer staging include MRI?. Nat Clin Pract Oncol.

[2] Houssami N, Solin LJ (2010). An appraisal of pre-operative MRI in breast cancer: more effective staging of the breast or much ado about nothing?. Maturitas.

[3] Jatoi I, Benson JR (2013). The case against routine preoperative breast MRI. Futur Oncol.

[4] Houssami N, Turner R, Macaskill P (2014). An individual person data meta-analysis of preoperative magnetic resonance imaging and breast cancer recurrence. J Clin Oncol.

[5] Houssami N, Turner RM, Morrow M (2017). Meta-analysis of pre-operative magnetic resonance imaging (MRI) and surgical treatment for breast cancer. Breast Cancer Res Treat.

[6] Lee J, Tanaka E, Eby PR (2017). Preoperative breast MRI: surgeons’ patient selection patterns and potential bias in outcomes analyses. AJR Am J Roentgenol.

[7] Newman LA (2020). Role of preoperative MRI in the management of newly diagnosed breast cancer patients. J Am Coll Surg.

[8] Lehman CD, Gatsonis C, Kuhl CK (2007). MRI evaluation of the contralateral breast in women with recently diagnosed breast cancer. N Engl J Med.

[9] Turnbull L, Brown S, Harvey I (2010). Comparative effectiveness of MRI in breast cancer (COMICE) trial: a randomised controlled trial. Lancet.

[10] Chou SHS, Romanoff J, Lehman CD (2021). Preoperative breast MRI for newly diagnosed ductal carcinoma in situ: imaging features and performance in a multicenter setting (ECOG-ACRIN E4112 Trial). Radiology.

[11] Peters NHGM, van Esser S, van den Bosch MAAJ (2011). Preoperative MRI and surgical management in patients with nonpalpable breast cancer: the MONET – randomised controlled trial. Eur J Cancer.

[12] Gonzalez V, Sandelin K, Karlsson A (2014). Preoperative MRI of the breast (POMB) influences primary treatment in breast cancer: a prospective, randomised, multicenter study. World J Surg.

[13] Arnaout A, Catley C, Booth CM (2015). Use of preoperative magnetic resonance imaging for breast cancer. JAMA Oncol.

[14] Wang SY, Long JB, Killelea BK (2016). Preoperative breast magnetic resonance imaging and contralateral breast cancer occurrence among older women with breast cancer. J Clin Oncol.

[15] Sardanelli F, Newstead GM, Putz B (2016). Gadobutrol-enhanced magnetic resonance imaging of the breast in the preoperative setting. Invest Radiol.

[16] Onega T, Zhu W, Weiss JE (2018). Preoperative breast MRI and mortality in older women with breast cancer. Breast Cancer Res Treat.

[17] Balleyguier C, Dunant A, Ceugnart L (2019). Preoperative breast magnetic resonance imaging in women with local ductal carcinoma in situ to optimize surgical outcomes: results from the randomised phase III trial IRCIS. J Clin Oncol.

[18] Pak LM, Banaag A, Koehlmoos TP, Nguyen LL, Learn PA (2020). Non-clinical drivers of variation in preoperative MRI utilization for breast cancer. Ann Surg Oncol.

[19] Sardanelli F, Boetes C, Borisch B (2010). Magnetic resonance imaging of the breast: recommendations from the EUSOMA working group. Eur J Cancer.

[20] Mann RM, Balleyguier C, Baltzer PA (2015). Breast MRI: EUSOBI recommendations for women’s information. Eur Radiol.

[21] Mann RM, Cho N, Moy L (2019). Breast MRI: state of the art. Radiology.

[22] Heller SL, Moy L (2019). MRI breast screening revisited. J Magn Reson Imaging.

[23] Mann RM, Athanasiou A, Baltzer PAT (2022). Breast cancer screening in women with extremely dense breasts recommendations of the European Society of Breast Imaging (EUSOBI). Eur Radiol.

[24] Berg WA (2022). Breast MRI for “the Masses”. Eur Radiol.

[25] Bakker MF, de Lange SV, Pijnappel RM (2019). Supplemental MRI screening for women with extremely dense breast tissue. N Engl J Med.

[26] Geuzinge HA, Bakker MF, Heijnsdijk EAM (2021). Cost-effectiveness of magnetic resonance imaging screening for women with extremely dense breast tissue. J Natl Cancer Inst.

[27] Comstock CE, Gatsonis C, Newstead GM (2020). Comparison of abbreviated breast MRI vs digital breast tomosynthesis for breast cancer detection among women with dense breasts undergoing screeninG. JAMA.

[28] Sardanelli F, Trimboli RM, Houssami N (2020). Solving the preoperative breast MRI conundrum: design and protocol of the MIPA study. Eur Radiol.

[29] Sardanelli F, Trimboli RM, Houssami N (2022). Magnetic resonance imaging before breast cancer surgery: results of an observational multicenter international prospective analysis (MIPA). Eur Radiol.

[30] Cohen E, Leung JWT (2018). Problem-Solving MR imaging for equivocal imaging findings and indeterminate clinical symptoms of the breast. Magn Reson Imaging Clin N Am.

[31] Fairbairn K, Cervantes A, Rayhrer C, Steen S (2020). Trends in contralateral prophylactic mastectomy. Aesthetic Plast Surg.

[32] Scheepens JCC, van Veer L, ’t, Esserman L, Belkora J, Mukhtar RA, (2021). Contralateral prophylactic mastectomy: a narrative review of the evidence and acceptability. Breast.

[33] Gommers JJ, Voogd AC, Broeders MJ (2021). Breast magnetic resonance imaging as a problem solving tool in women recalled at biennial screening mammography: a population-based study in the Netherlands. Breast.

[34] Taskin F, Polat Y, Erdogdu IH, Turkdogan FT, Ozturk VS, Ozbas S (2018). Problem-solving breast MRI: useful or a source of new problems?. Diagnostic Interv Radiol.

[35] Schmidt MK, Kelly JE, Brédart A (2023). EBCC-13 manifesto: Balancing pros and cons for contralateral prophylactic mastectomy. Eur J Cancer.

[36] Cozzi A, Magni V, Zanardo M, Schiaffino S, Sardanelli F (2022). Contrast-enhanced mammography: a systematic review and meta-analysis of diagnostic performance. Radiology.

[37] European Commission Initiative on Breast Cancer (2022) Planning surgical treatment: contrast-enhanced spectral mammography. https://healthcare-quality.jrc.ec.europa.eu/european-breast-cancer-guidelines/surgical-planning/CESM. Accessed 15 Jan 2023

[38] Åhsberg K, Gardfjell A, Nimeus E, Ryden L, Zackrisson S (2021). The PROCEM study protocol: added value of preoperative contrast-enhanced mammography in staging of malignant breast lesions - a prospective randomised multicenter study. BMC Cancer.

