# Supplementary material for: Screening and diagnostic breast MRI: how do they impact surgical treatment? Insights from the MIPA study
Source: Eur Radiol. 2023 May 3;33(9):6213–25. doi: 10.1007/s00330-023-09600-5 (PMC10415233; doi:10.1007/s00330-023-09600-5)
Supplement: Supplementary file 1 — Supplementary file1 (PDF 285 KB) [file 330_2023_9600_MOESM1_ESM.pdf]

Supplementary Material

Demographic and clinical data

As detailed in Table E1, median patient age among subgroups matched routine MRI practice [1], with the noMRI subgroup having a significantly (adjusted $p < 0.001$) higher median age than all MRI subgroups (62 years, IQR 52–70 years): among them, earlier MRI referral implied a younger age, with the S-MRI subgroup having a significantly (adjusted $p < 0.001$) lower median age (52 years, IQR 45–58) than the P-MRI subgroup (median 57 years, IQR 49–65) and the D-MRI subgroup being halfway between them (54 years, IQR 47–64), closer to the S-MRI subgroup (adjusted $p = 0.081$) than to the P-MRI subgroup (adjusted $p < 0.001$).

This scenario was mirrored both by breast density and hormonal status distributions. S-MRI patients had the highest percentage of dense breasts (62% with ACR *c* and *d* classes, compared to 49% among D-MRI patients and 50% among P-MRI patients). While no significant overall difference in density distribution was seen among the MRI subgroups (adjusted p values > 0.784), their cumulative percentages of dense breasts (62% in the S-MRI subgroup, 49% in the D-MRI subgroup, 50% in the P-MRI subgroup) were significantly higher (adjusted $p < 0.001$ for all comparisons) than the 33% of the noMRI subgroup. Likewise, the cumulative percentage of pre- and peri-menopausal patients reached 54% in the S-MRI subgroup, decreasing to 48% (D-MRI, adjusted $p = 0.778$ versus S-MRI), 39% (P-MRI, adjusted $p = 0.019$ versus S-MRI), and 27% in the noMRI subgroup (adjusted $p < 0.001$ for all comparisons).

As expected, no differences were observed between the D-MRI, P-MRI, and noMRI subgroup in terms of the number of patients with high familial risk (3 or more relatives with breast cancer history) or with proven *BRCA1*/*2* mutations, as none of these characteristics was found in more than 1% of patients in each of these subgroups. Conversely, 10% (11/113) of patients in the S-MRI

subgroup had high familial risk and 30% (32/105) had a proven *BRCA1* (15%) or *BRCA2* (15%) mutation ($p < 0.001$).

Imaging

As reported in Table E2, information from conventional imaging was obviously more commonly available in the P-MRI and noMRI subgroups, the P-MRI subgroup having significantly higher rates of patients with cancers with an imaging maximal diameter larger than 20 mm (40% versus 33% at mammography, 31% versus 24% at ultrasonography, respectively) and of patients with multifocal and multicentric cancers (14% versus 9% at mammography, 19% versus 9% at ultrasonography, respectively, adjusted $p < 0.001$ for all comparisons). Sample paucity and imbalance did not allow to highlight significant pairwise differences in conventional imaging features among the other MRI subgroups, but a relative polarisation of the S-MRI subgroup towards high rates of patients with unifocal (93% versus 87% and 86% at mammography in the D-MRI and P-MRI subgroups, respectively) and small cancers (20% versus 27% and 31% at ultrasonography in the D-MRI and P-MRI subgroups, respectively) can be observed. While no significant difference ($p = 0.017$) was observed among MRI subgroups in terms of cancer focality at MRI (79% unifocal cancers in the S-MRI subgroup versus 71% and 65% in the D-MRI and P-MRI subgroups, respectively), the rate of cancers larger than 20 mm was markedly lower in the S-MRI (30%) and D-MRI (40%) subgroups (non-significant pairwise comparison with adjusted $p = 0.271$), compared to 51% in the P-MRI subgroup (adjusted $p < 0.001$ for both comparisons).

Biopsy and surgical pathology

Comparisons among the subgroups for three indicators from core-needle or vacuum-assisted biopsy (CNB/VAB) and six indicators from surgical pathology are detailed in Table E3 and E4. At biopsy, no significant difference was observed in the rates of occurrence of pure ductal carcinoma in situ (DCIS) among the four subgroups ($p = 0.192$ at CNB/VAB, $p = 0.012$ at pathology), the D-MRI

and P-MRI subgroups always exhibiting marginally lower rates (15% and 17% at CNB/VAB, respectively, both 14% at surgical pathology) than the S-MRI and noMRI subgroups (21% and 18% at CNB/VAB, 18% and 17% at surgical pathology, respectively).

The rates of the association of DCIS to invasive cancer were instead significantly different among subgroups both at CNB/VAB and surgical pathology ($p < 0.001$ for both comparisons). At biopsy, this comparison was dominated by the high rate of associated DCIS in the D-MRI subgroup compared to the P-MRI subgroup (37% versus 31%, respectively, adjusted $p = 0.003$), while subgroup size imbalance hindered the emergence of a significant difference between the 41% rate in the S-MRI subgroup and other subgroups (adjusted $p \geq 0.114$ in all comparisons). At surgical pathology, all rates markedly increased and the comparison was dominated by the difference between the 73% rate of DCIS association to invasive cancers in the S-MRI subgroup compared to other subgroups (adjusted p values ≤ 0.010).

The presence of invasive lobular carcinoma—as an exclusive entity or as a component in mixed ductal and lobular cancers—significantly differed among subgroups both at CNB/VAB ($p < 0.001$) and surgical pathology ($p < 0.001$). Both comparisons were led by the higher rates of invasive lobular carcinoma occurrence in the D-MRI and P-MRI subgroups (15% and 17% at CNB/VAB, 21% and 22% at surgical pathology, respectively, adjusted p values < 0.001) compared to the S-MRI and noMRI subgroups (10% and 8% at CNB/VAB, 13% and 12% at surgical pathology, respectively).

As already observed at conventional and MRI imaging, the D-MRI and P-MRI subgroups had significantly lower rates of unifocal cancers (77% for both subgroups) compared to the noMRI subgroup (89%, overall and adjusted p values < 0.001), while a similar non-significant trend was also seen for the S-MRI subgroup (83% rate of unifocal cancers, adjusted p values ≥ 0.327).

Likewise, the rate of cancers larger than 20 mm was again significantly lower in the S-MRI (28%) and noMRI (27%) subgroups compared to the D-MRI (35%) and P-MRI (42%) subgroups, respectively (adjusted p values ≤ 0.021).

Multivariable model building

As detailed in the Methods section, variable selection for multivariable logistic regression was performed using stepwise multivariable linear regression (forward selection with $p < 0.1$ as the threshold for variable inclusion), taking into account a clinically-reasoned pool of variables for each of the four models, each focused on a specific surgical endpoint. Of note, only those information that were effectively available to the multidisciplinary meeting when planning each specific surgical stage were considered for inclusion in the model. More specifically, in the two models investigating first-line mastectomy (Table E5) and bilateral first-line mastectomy (Table E6), variables entering multivariable linear regression were those from demographic, imaging, and CNB/VAB data. In the model investigating reoperation (Table E7), CNB/VAB data were replaced by surgical pathology data from the first surgical procedure. Finally, the model investigating the overall occurrence of mastectomy (Table E8) was built drawing from all aforementioned data categories.

References

1. Lee J, Tanaka E, Eby PR et al (2017) Preoperative Breast MRI: Surgeons' Patient Selection Patterns and Potential Bias in Outcomes Analyses. Am J Roentgenol 208:923–932. doi:10.2214/AJR.16.17038.

Tables

Table E1 Demographic and clinical characteristics of patients enrolled in the three MRI subgroups and in the noMRI subgroup.

**Subgroup**		**Screening (S-MRI)**	**Diagnostic (D-MRI)**	**Preoperative (P-MRI)**	**noMRI**	**P values for comparisons**	
**Patients**		**114**	**510**	**2441**	**2763**	**Overall**	**Group-wise**
**Median age (interquartile range)**		52 (45–58)	54 (47–64)	57 (49–65)	62 (52–70)	< 0.001	S-MRI vs D-MRI 0.081<br>S-MRI vs P-MRI < 0.001<br>S-MRI vs noMRI < 0.001<br>D-MRI vs P-MRI < 0.001<br>D-MRI vs noMRI < 0.001<br>P-MRI vs noMRI < 0.001
**Breast density**	*Known*	*96*	*483*	*2336*	*2503*	< 0.001	S-MRI vs D-MRI 0.909<br>S-MRI vs P-MRI 0.784<br>S-MRI vs noMRI < 0.001<br>D-MRI vs P-MRI 1.000<br>D-MRI vs noMRI < 0.001<br>P-MRI vs noMRI < 0.001
	ACR BI-RADS class *a*	9 (9%)	56 (12%)	240 (10%)	513 (21%)		
	ACR BI-RADS class *b*	28 (29%)	187 (39%)	925 (40%)	1159 (46%)		
	ACR BI-RADS class *c*	40 (42%)	176 (36%)	846 (36%)	707 (28%)		
	ACR BI-RADS class *d*	19 (20%)	64 (13%)	325 (14%)	124 (5%)		
**Familial risk***	*Known*	*113*	*509*	*2432*	*2749*	< 0.001	S-MRI vs D-MRI < 0.001<br>S-MRI vs P-MRI < 0.001<br>S-MRI vs noMRI < 0.001<br>D-MRI vs P-MRI 1.000<br>D-MRI vs noMRI 1.000<br>P-MRI vs noMRI 1.000
	No	102 (90%)	503 (99%)	2397 (99%)	2715 (99%)		
	Yes	11 (10%)	6 (1%)	35 (1%)	34 (1%)		
**Proven *BRCA1* or *BRCA2* mutation**	*Known*	*105*	*501*	*2395*	*2715*	< 0.001	S-MRI vs D-MRI < 0.001<br>S-MRI vs P-MRI < 0.001<br>S-MRI vs noMRI < 0.001<br>D-MRI vs P-MRI 1.000<br>D-MRI vs noMRI 1.000<br>P-MRI vs noMRI 0.966
	No	73 (70%)	496 (99%)	2375 (99%)	2701 (99%)		
	Yes	32 (30%)	5 (1%)	20 (1%)	14 (1%)		
**Hormonal status**	*Known*	*113*	*508*	*2432*	*2748*	< 0.001	S-MRI vs D-MRI 0.778<br>S-MRI vs P-MRI 0.019<br>S-MRI vs noMRI < 0.001<br>D-MRI vs P-MRI 0.092<br>D-MRI vs noMRI < 0.001<br>P-MRI vs noMRI < 0.001
	Premenopausal	51 (45%)	176 (35%)	720 (30%)	484 (18%)		
	Perimenopausal	10 (9%)	66 (13%)	252 (10%)	247 (9%)		
	Postmenopausal	52 (46%)	266 (52%)	1460 (60%)	2017 (73%)		

*Three or more relatives with breast cancer

ACR BI-RADS, American College of Radiology Breast Imaging Data and Reporting System.

Table E2 Imaging features at conventional imaging and MRI among the four patient subgroups.

**Subgroup**		**Screening (S-MRI)**	**Diagnostic (D-MRI)**	**Preoperative (P-MRI)**	**noMRI**	**P values for comparisons**	
**Patients**		**114**	**510**	**2441**	**2763**	**Overall**	**Group-wise**
**Lesion focus at DM**	*Known*	*61*	*384*	*2143*	*2435*	< 0.001	S-MRI vs D-MRI 1.000<br>S-MRI vs P-MRI 1.000<br>S-MRI vs noMRI 1.000<br>D-MRI vs P-MRI 1.000<br>D-MRI vs noMRI 0.150<br>P-MRI vs noMRI < 0.001
	Unifocal	57 (94%)	334 (87%)	1843 (86%)	2223 (91%)		
	Multifocal	2 (3%)	38 (10%)	214 (10%)	164 (7%)		
	Multicentric	2 (3%)	12 (3%)	86 (4%)	48 (2%)		
**Lesion focus at US**	*Known*	*58*	*373*	*2051*	*2179*	< 0.001	S-MRI vs D-MRI 0.262<br>S-MRI vs P-MRI 0.195<br>S-MRI vs noMRI 1.000<br>D-MRI vs P-MRI 1.000<br>D-MRI vs noMRI < 0.001<br>P-MRI vs noMRI < 0.001
	Unifocal	55 (95%)	307 (82%)	1671 (81%)	1986 (91%)		
	Multifocal	2 (3%)	58 (16%)	301 (15%)	151 (7%)		
	Multicentric	1 (2%)	8 (2%)	79 (4%)	42 (2%)		
**Lesion focus at MRI**	*Known*	*81*	*443*	*2249*	–	0.017	S-MRI vs D-MRI 0.803<br>S-MRI vs P-MRI 0.122<br>D-MRI vs P-MRI 0.132
	Unifocal	64 (79%)	313 (71%)	1472 (65%)	–		
	Multifocal	11 (14%)	93 (21%)	507 (23%)	–		
	Multicentric	6 (7%)	37 (8%)	270 (12%)	–		
**Largest lesion diameter at DM**	*Known*	*64*	*364*	*2089*	*2411*	< 0.001	S-MRI vs D-MRI 1.000<br>S-MRI vs P-MRI 1.000<br>S-MRI vs noMRI 1.000<br>D-MRI vs P-MRI 0.031<br>D-MRI vs noMRI 1.000<br>P-MRI vs noMRI < 0.001
	< 20 mm	41 (64%)	245 (67%)	1243 (60%)	1610 (67%)		
	≥ 20 mm	23 (36%)	119 (33%)	846 (40%)	801 (33%)		
**Largest lesion diameter at US**	*Known*	*74*	*374*	*2068*	*2205*	< 0.001	S-MRI vs D-MRI 1.000<br>S-MRI vs P-MRI 0.423<br>S-MRI vs noMRI 1.000<br>D-MRI vs P-MRI 0.849<br>D-MRI vs noMRI 1.000<br>P-MRI vs noMRI < 0.001
	< 20 mm	59 (80%)	274 (73%)	1436 (69%)	1677 (76%)		
	≥ 20 mm	15 (20%)	100 (27%)	632 (31%)	528 (24%)		
**Largest lesion diameter at MRI**	*Known*	*102*	*449*	*2281*	–	< 0.001	S-MRI vs D-MRI 0.271<br>S-MRI vs P-MRI < 0.001<br>D-MRI vs P-MRI < 0.001
	< 20 mm	71 (70%)	271 (60%)	1115 (49%)	–		
	≥ 20 mm	31 (30%)	178 (40%)	1166 (51%)	–		

DM, digital mammography; US, ultrasonography; MRI, magnetic resonance imaging.

Table E3 Core-needle or vacuum-assisted biopsy and surgical pathology characteristics among the four patient subgroups.

**Subgroups**		**Screening (S-MRI)**	**Diagnostic (D-MRI)**	**Preoperative (P-MRI)**	**noMRI**	**P values for comparisons**	
**Patients**		**114**	**510**	**2441**	**2763**	**Overall**	**Group-wise**
**Malignancy type**	*Unknown*	*7*	*49*	*74*	*215*	0.192	S-MRI vs D-MRI 0.656<br>S-MRI vs P-MRI 1.000<br>S-MRI vs noMRI 1.000<br>D-MRI vs P-MRI 1.000<br>D-MRI vs noMRI 0.450<br>P-MRI vs noMRI 1.000
	Invasive cancer	84 (79%)	392 (85%)	1959 (83%)	2078 (82%)		
	Pure DCIS	23 (21%)	69 (15%)	408 (17%)	470 (18%)		
**DCIS associated to invasive cancer**	No	41 (59%)	225 (63%)	1321 (72%)	1273 (69%)	< 0.001	S-MRI vs D-MRI 1.000<br>S-MRI vs P-MRI 0.120<br>S-MRI vs noMRI 0.483<br>D-MRI vs P-MRI 0.003<br>D-MRI vs noMRI 0.114<br>P-MRI vs noMRI 0.307
	Yes	28 (41%)	132 (37%)	507 (28%)	563 (31%)		
**Lobular component**	No	76 (90%)	332 (85%)	1618 (83%)	1914 (92%)	< 0.001	S-MRI vs D-MRI 1.000<br>S-MRI vs P-MRI 0.361<br>S-MRI vs noMRI 1.000<br>D-MRI vs P-MRI 1.000<br>D-MRI vs noMRI < 0.001<br>P-MRI vs noMRI < 0.001
	Yes	8 (10%)	60 (15%)	341 (17%)	164 (8%)		

DCIS, ductal carcinoma in situ; MRI, magnetic resonance imaging.

Table E4 Surgical pathology characteristics among the four patient subgroups.

**Subgroups**		**Screening (S-MRI)**	**Diagnostic (D-MRI)**	**Preoperative (P-MRI)**	**noMRI**	**P values for comparisons**	
**Patients**		**114**	**510**	**2441**	**2763**	**Overall**	**Group-wise**
**Malignancy type**	*No residual tumor*	*9*	*67*	*267*	*355*	0.011	S-MRI vs D-MRI 1.000<br>S-MRI vs P-MRI 1.000<br>S-MRI vs noMRI 1.000<br>D-MRI vs P-MRI 1.000<br>D-MRI vs noMRI 0.631<br>P-MRI vs noMRI 0.010
	Invasive cancer	86 (82%)	380 (86%)	1870 (86%)	1990 (83%)		
	Pure DCIS	19 (18%)	63 (14%)	304 (14%)	418 (17%)		
**DCIS associated to invasive cancer**	No	23 (27%)	195 (51%)	821 (44%)	873 (44%)	< 0.001	S-MRI vs D-MRI < 0.001<br>S-MRI vs P-MRI 0.010<br>S-MRI vs noMRI 0.010<br>D-MRI vs P-MRI 0.049<br>D-MRI vs noMRI 0.046<br>P-MRI vs noMRI 1.000
	Yes	63 (73%)	185 (49%)	1049 (56%)	1117 (56%)		
**Lobular component**	No	75 (87%)	300 (79%)	1454 (78%)	1760 (88%)	< 0.001	S-MRI vs D-MRI 0.814<br>S-MRI vs P-MRI 0.320<br>S-MRI vs noMRI 1.000<br>D-MRI vs P-MRI 1.000<br>D-MRI vs noMRI < 0.001<br>P-MRI vs noMRI < 0.001
	Yes	11 (13%)	80 (21%)	416 (22%)	230 (12%)		
**Lesion focus**	*Known*	*103*	*442*	*2142*	*2384*	< 0.001	S-MRI vs D-MRI 1.000<br>S-MRI vs P-MRI 0.327<br>S-MRI vs noMRI 0.782<br>D-MRI vs P-MRI 0.341<br>D-MRI vs noMRI < 0.001<br>P-MRI vs noMRI < 0.001
	Unifocal	86 (83%)	342 (77%)	1640 (76%)	2119 (89%)		
	Multifocal	15 (15%)	76 (17%)	319 (15%)	209 (9%)		
	Multicentric	2 (2%)	24 (6%)	183 (9%)	56 (2%)		
**Largest lesion diameter**	*Known*	*101*	*355*	*1886*	*1953*	< 0.001	S-MRI vs D-MRI 0.879<br>S-MRI vs P-MRI 0.021<br>S-MRI vs noMRI 1.000<br>D-MRI vs P-MRI 0.090<br>D-MRI vs noMRI 0.013<br>P-MRI vs noMRI < 0.001
	< 20 mm	73 (72%)	229 (65%)	1086 (58%)	1416 (73%)		
	≥ 20 mm	28 (28%)	126 (35%)	800 (42%)	537 (27%)		

DCIS, ductal carcinoma in situ; MRI, magnetic resonance imaging.

Table E5 Multivariable linear stepwise regression for first-line mastectomy. Criterion: probability of F-to-remove ≥ 0.100.

			**Standardized β**	**t**	***p* value**
	Constant	–	–	*-1.131*	*0.258*
**Subgroup**	MRI referral	Included step 2	0.130	10.104	< 0.001
**Clinical characteristics**	Breast density	Included step 8	0.060	4.381	< 0.001
	Familial risk of breast cancer	Included step 10	0.042	3.392	0.001
	Hormonal status	Included step 5	-0.096	-7.021	< 0.001
**Imaging**	Lesion focus at DM	Included step 6	0.075	5.192	< 0.001
	Largest lesion diameter at DM ≥ 20 mm	Included step 4	0.116	7.392	< 0.001
	Lesion focus at US	Included step 3	0.145	9.468	< 0.001
	Largest lesion diameter at US ≥ 20 mm	Included step 1	0.120	7.610	< 0.001
**Core-needle or vacuum-assisted biopsy**	Pure DCIS	Included step 9	0.050	3.632	< 0.001
	DCIS associated to invasive cancer	Excluded	0.007	0.572	0.567
	Lobular component	Included step 7	0.071	5.589	< 0.001

Durbin–Watson statistic: 1.791. All included variables are as described in Tables E1, E2, E3.

MRI, magnetic resonance imaging; DM, digital mammography; US, ultrasonography; DCIS, ductal carcinoma in situ.

Table E6 Multivariable linear stepwise regression for first-line bilateral mastectomy. Criterion: probability of F-to-remove ≥ 0.100.

			Standardized β	t	*p* value
	Constant	–	–	*0.724*	*0.469*
**Subgroup**	MRI referral	Included step 1	0.079	5.742	< 0.001
**Clinical characteristics**	Breast density	Excluded	0.021	1.391	0.164
	Familial risk of breast cancer	Included step 2	0.069	5.159	< 0.001
	Hormonal status	Included step 5	-0.037	-2.757	0.006
**Imaging**	Lesion focus at DM	Excluded	0.022	1.498	0.134
	Largest lesion diameter at DM ≥ 20 mm	Excluded	-0.012	-0.899	0.369
	Lesion focus at US	Included step 3	0.060	4.415	< 0.001
	Largest lesion diameter at US ≥ 20 mm	Excluded	-0.011	-0.808	0.419
**Core-needle or vacuum-assisted biopsy**	Pure DCIS	Excluded	-0.012	-0.830	0.407
	DCIS associated to invasive cancer	Excluded	0.017	1.277	0.202
	Lobular component	Included step 4	0.052	3.849	< 0.001

Durbin–Watson statistic: 2.033. All included variables are as described in Tables E1, E2, E3

MRI, magnetic resonance imaging; DM, digital mammography; US, ultrasonography; DCIS, ductal carcinoma in situ.

Table E7 Multivariable linear stepwise regression for reoperation for close or positive margins. Criterion: probability of F-to-remove ≥ 0.100.

			**Standardized β**	**t**	***p* value**
	Constant	–	–	*4.491*	*< 0.001*
**Subgroup**	MRI referral	Included step 3	-0.062	-4.534	< 0.001
**Clinical characteristics**	Breast density	Excluded	0.023	1.566	0.117
	Familial risk of breast cancer	Excluded	0.014	1.084	0.278
	Hormonal status	Included step 7	0.040	2.966	0.003
**Imaging**	Lesion focus at DM	Excluded	0.005	0.332	0.740
	Largest lesion diameter at DM ≥ 20 mm	Excluded	-0.008	-0.493	0.622
	Lesion focus at US	Included step 5	-0.048	-3.247	0.001
	Largest lesion diameter at US ≥ 20 mm	Included step 9	-0.031	-2.051	0.040
**Surgical pathology**	Pure DCIS	Included step 1	0.094	6.126	< 0.001
	DCIS associated to invasive cancer	Included step 2	0.086	5.797	< 0.001
	Lobular component	Included step 6	0.037	2.659	0.008
	Multifocal or multicentric cancer	Included step 4	0.056	3.926	< 0.001
	Largest lesion diameter ≥ 20 mm	Included step 8	0.051	3.400	0.001

Durbin–Watson statistic: 1.882. All included variables are as described in Tables E1, E2, E3.

MRI, magnetic resonance imaging; DM, digital mammography; US, ultrasonography; DCIS, ductal carcinoma in situ.

Table E8 Multivariable linear stepwise regression for first- and second-line (overall) mastectomy. Criterion: probability of F-to-remove ≥ 0.100.

			**Standardized β**	**t**	***p* value**
	Constant	–	–	*0.099*	*0.921*
**Subgroup**	MRI referral	Included step 3	0.095	7.603	< 0.001
**Clinical characteristics**	Breast density	Included step 10	0.059	4.465	< 0.001
	Familial risk of breast cancer	Included step 12	0.045	3.723	< 0.001
	Hormonal status	Included step 4	-0.085	-6.502	< 0.001
**Imaging**	Lesion focus at DM	Included step 11	0.056	3.976	< 0.001
	Largest lesion diameter at DM ≥ 20 mm	Included step 5	0.089	5.733	< 0.001
	Lesion focus at US	Included step 6	0.087	5.813	< 0.001
	Largest lesion diameter at US ≥ 20 mm	Included step 2	0.098	6.293	< 0.001
**Core-needle or vacuum-assisted biopsy**	Pure DCIS	Included step 8	0.081	4.766	< 0.001
	DCIS associated to invasive cancer	Excluded	0.017	1.316	0.188
	Lobular component	Included step 9	0.051	4.060	< 0.001
**Surgical pathology**	Pure DCIS	Included step 13	-0.054	-3.126	0.002
	DCIS associated to invasive cancer	Included step 14	-0.032	-2.371	0.018
	Lobular component	Excluded	0.018	0.739	0.460
	Multifocal or multicentric cancer	Included step 1	0.244	18.915	< 0.001
	Largest lesion diameter ≥ 20 mm	Included step 7	0.097	7.004	< 0.001

Durbin–Watson statistic: 1.849. All included variables are as described in Tables E1, E2, E3.

MRI, magnetic resonance imaging; DM, digital mammography; US, ultrasonography; DCIS, ductal carcinoma in situ.
